# Maternal and neonatal outcomes with the use of long acting, compared to intermediate acting basal insulin (NPH) for managing diabetes during pregnancy: a systematic review and meta-analysis

**DOI:** 10.1186/s13098-022-00925-7

**Published:** 2022-10-21

**Authors:** Jijiao Wang, Xiaochen Ji, Ting Liu, Nan Zhao

**Affiliations:** The Second Affiliated Hospital of Dalian Medical University, No 467, Zhongshan Road, Dalian, 116021 Liaoning Province China

**Keywords:** Intermediate acting insulin, Neutral protamine Hagedron, NPH, Long-acting insulin analogues, Glargine, Detemir, Obstetric outcomes, Neonatal outcomes, Complications, Meta-analysis

## Abstract

**Background:**

To assess the impact of long-acting insulin analogues, compared to intermediate acting neutral protamine Hagedron (NPH), on maternal, perinatal and neonatal outcomes.

**Methods:**

Studies for inclusion in the review were identified using a structured search strategy in PubMed, Scopus and Cochrane Central Register of Controlled Trials (CENTRAL) database. Studies that were randomized controlled trials or observational in design were considered for inclusion. Eligible studies should have compared the maternal, perinatal and neonatal outcomes between pregnant women with gestational diabetes mellitus (GDM) managed by intermediate acting (NPH) and by long-acting insulin analogues. Statistical analysis was performed using STATA software.

**Results:**

We found 17 studies to be eligible for inclusion. The mean gestational weight gain and risk of maternal hypoglycaemia, hypertensive disorder, caesarean delivery, spontaneous abortion, endometritis and wound infection or dehiscence were similar among pregnant women with GDM managed using long-acting insulin analogues and NPH. Those receiving long-acting insulin analogues had significantly lower HbA1c values in the second (WMD − .09, 95% CI 0.12, − 0.06; N = 4) and third trimester (WMD − 0.08, 95% CI − 0.14, − 0.02; N = 12). The mean gestational age and birth weight and risk of perinatal mortality, prematurity, large for gestational age, small for gestational age, shoulder dystocia and congenital abnormalities was similar among babies in both groups. No statistically significant differences in risk of admission to neonatal intensive care unit, respiratory distress, neonatal hypoglycaemia, 5 min APGAR score of < 7, neonatal hyperbilirubinemia and sepsis was observed. The quality of pooled evidence, as per GRADE criteria, was judged to be “very low” for all the maternal and neonatal outcomes considered.

**Conclusions:**

Findings suggest no significant differences in the maternal, perinatal and neonatal outcomes between intermediate and long-acting insulin analogues. The results provide support for use of long-acting insulin analogues in women with GDM. However, evidence is still needed from high quality randomized controlled trials to arrive at a recommendation for inclusion in routine clinical care.

**Supplementary Information:**

The online version contains supplementary material available at 10.1186/s13098-022-00925-7.

## Background

The prevalence of diabetes in pregnancy is increasing and it is estimated that around 8–10% of the pregnancies, globally are affected by gestational diabetes (GDM) [1, 2]. In the late pregnancy, there is increased production of diabetogenic hormones, such as human placental lactogen (HPL), by placenta and this leads to insulin resistance [3, 4]. There is a concurrent hyper functionality of the beta-cells, however, it fails to counteract the insulin resistance and therefore, it leads to GDM [3, 4]. It is well documented that GDM not only leads to adverse maternal, perinatal and neonatal outcomes but also increases the risk of later obesity and diabetes in both the mother and the child [5–10]. Some of the well-known complications of gestational diabetes include enhanced maternal risk of hypertensive disorder in mother, delivery by cesarean section and traumatic delivery [7, 8]. For the child, the risks include preterm delivery, perinatal death, hypoglycemia, respiratory distress, congenital malformations and admission to a neonatal intensive care unit [5, 7, 8].

Improved glycemic control during pregnancy could possibly avert many of these adverse outcomes [11]. The current clinical management guidelines therefore aim at optimal glycemic control. One of the preferred options for management of GDM is human insulin as it does not cross the placental barrier but there are certain limitations to this [12, 13]. There is an associated risk of hypoglycemia and fluctuations in blood glucose levels [12, 13]. These limitations could be avoided through use of insulin analogues that have a more sustained action with no sharp peaks [14–17]. Use of basal insulin (neutral protamine Hagedron, NPH; intermediate acting) as well as insulin analogues (glargine, detemir and degludec; long acting) tend to curb the excessive glucose production by liver, are long acting and require administration only once or twice in a day [14–17]. The critical difference between NPH and long-acting insulin analogues is in terms of their pharmacodynamics. NPH tends to have achieve peak at 4–12 h post-administration and the total duration of action is around 14–15 h [14, 16, 17]. On the other hand, long-acting analogues are more sustained in duration of action i.e., around 20–24 h and they do not have sharp peak and fall [14, 16, 17]. Therefore, the risk of glycemic fluctuations and hypoglycemia might be minimized. Based on this important difference in the pharmacodynamics, it is thought that these newer long-acting analogues may offer better glycemic control and consequently better pregnancy and child outcomes.

Recently, there have been an upsurge in studies that have assessed the efficacy of long-acting insulin preparations in managing GDM. Three systematic reviews had earlier attempted to synthesize evidence from studies comparing maternal and perinatal outcomes among women managed with long-acting insulin preparations and intermediate acting NPH insulin [18–20]. All of these reviews indicated that there are no significant differences in maternal, perinatal or neonatal outcomes in women with GDM treated with either NPH or long-acting insulin analogues. However, these reviews were conducted more than half a decade earlier. With more studies being published on this issue, there is a need for an updated systematic review. The current meta-analysis aims to synthesize findings from studies comparing maternal, perinatal and neonatal outcomes in pregnant women with GDM managed by long and intermediate acting insulin formulations.

## Materials and methods

### Search strategy

The study was designed by two authors (JW & XJ). The search engines used for identification of relevant studies were PubMed, Scopus, and Cochrane Central Register of Controlled Trials (CENTRAL). Two authors were involved in the literature search independent of each other (JW & TL). The intent was to identify studies published in English language, prior to 30th September 2021, using a thorough search strategy. We did a second round of search of these databases to identify possible new studies published between our previous search and 30th August 2022. The search strategies used for the three databases have been presented in Additional file 1: Table S1. This literature search targeted studies examining maternal, perinatal, and neonatal outcomes for pregnant women with GDM managed either by intermediate acting (NPH) or long-acting insulin analogues (glargine, detemir and degludec). For conducting this meta-analysis, we followed the PRISMA guidelines and had registered the protocol at International Prospective Registry of Systematic Reviews (PROSPERO; registration number-CRD42021282751) [21].

### Selection criteria and methods

After executing the search strategy in the above listed database, the first step was to remove the duplicates. Thereafter, all unique studies were independently reviewed by two study authors. The initial review was focused on title and abstracts. This was followed by full texts of studies that seemed to be eligible for inclusion in the review. In instances where there were disagreements related to selection of a study, the two authors discussed and arrived at a mutual consensus. The reference lists of the selected studies were also reviewed to identify additional studies for inclusion.

### Inclusion criteria and exclusion criteria

It was decided *apriori* to included studies that were either randomized controlled trials (RCTs) or observational in design i.e., case-control and cohort. For a study to be eligible for inclusion, it should have been conducted in pregnant women with GDM and had compared outcomes of interest among those that were managed using neutral protamine Hagedron (NPH) and those managed with long-acting insulin analogues.

We excluded case reports and reviews. Studies that did not report on the outcomes of interest or did not provide comparative findings based on the comparison groups of interest were excluded.

### Data extraction and quality assessment

The relevant data from the included studies were extracted by two authors independently using a pre-tested data extraction sheet (JW & TL). The quality of the studies was evaluated independently by the two authors using standardized tools i.e., Revised Cochrane ‘Risk of bias’ tool for randomised trials (RoB 2) and Newcastle-Ottawa Quality Assessment Scale (NOS) for observational studies [22, 23]. We used the RoB 2 Excel tool to implement RoB 2 (available from https://www.riskofbias.info/) and produced risk of bias plots using “robvis” tool [24].

### Statistical analysis

The findings of the included studies were pooled to provide effect sizes either as relative risk (RR) for categorical outcomes or as weighted mean difference (WMD) for continuous outcomes. We reported the pooled effect sizes along with 95% confidence intervals (CI). Depending upon the degree of heterogeneity (denoted by I^2^), we used either random effects model (I^2^ > 40%) or fixed effects model (I^2^ ≤ 40%) [25]. A subgroup analysis was conducted based on the type of long-acting insulin analogue used. For the purpose of denoting statistical significance, a P-value of less than 0.05 was considered. Presence of publication bias was assessed using Egger’s test and visually inspected using funnel plots [26]. We conducted all analysis using STATA software version 16.0. The quality of pooled evidence obtained was assessed using the GRADE criteria [27]. Data analysis was conducted by two authors (JW & XJ).

## Results

**Fig. 1:**
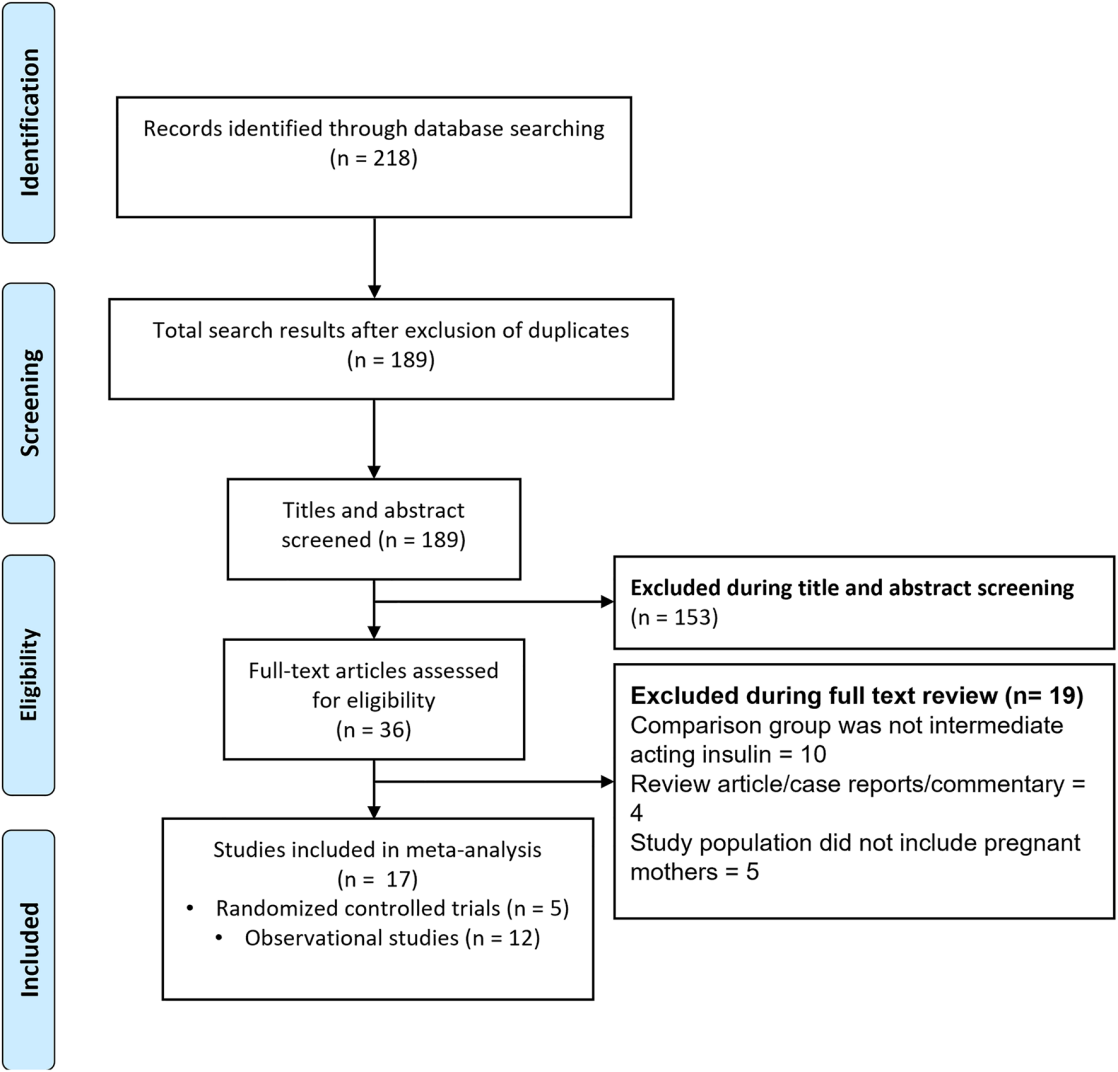
Selection process of the studies included in the review

We identified 189 unique citations upon executing the search strategy and eliminating the duplicates (Fig. 1). Title and abstract screening eliminated 153 citations, with 19 more excluded by full-text review. This left 17 studies for inclusion (Table 1) [28–44]. Five studies were randomized controlled trials (RCTs) and six were retrospective cohort-based studies. Three studies were case-control in design and two were retrospective review of medical records. One included study was prospective cohort in design. Seven studies were done in USA, three in Italy and two were multicentric. One study each was done in Spain, China, Brazil, Finland and United Kingdom. Out of the 17 included studies, nine studies used “glargine” as a long-acting insulin analogue and six studies used “detemir”. Remaining two studies has a mix study population with some using glargine and other using determir.

**Table 1:** Characteristics of the studies included in the meta-analysis

Author (year of publication)	Study design	Country	Participant characteristics	Long acting insulin used	Sample size	Key outcomes (comparison group: intermediate acting insulin)
Bartal et al. (2021) [28]	RCT	USA	Women with singleton gestation and type 2 diabetes; gestational diabetes mellitus (GDM) diagnosed before 20 weeks gestation; mean age of 32 years and 88% multiparous; pre-pregnancy BMI of > 30 kg/m2 (83%); mean gestational age at randomization (13 wks)	Detemir	108 (57-Detemir; 51- neutral protamine Hagedorn)	Maternal outcomes Symptomatic hypoglycaemia: RR 0.33 (95% CI 0.13, 0.76) Hypertensive disorder: RR 0.81 (95% CI 0.54, 1.16) Mean (SD) gestational weight gain (kg): 7 (1.83) vs. 9 (1.78) Caesarean section: RR 0.99 (95% CI 0.73, 1.34) Postpartum haemorrhage: RR 1.25 (95% CI 0.50, 3.26) Endometritis: RR 0.80 (95% CI 0.22, 2.91) Wound infection or dehiscence: RR 0.83 (95% CI 0.26, 2.64) Postpartum readmission: RR 1.71 (95% CI 0.63, 5.01) Hospitalization for glucose control: RR 0.89 (95% CI 0.42, 1.87) Induction of labour: RR 0.96 (95% CI 0.66, 1.37) Foetal/neonatal outcomes Mean (SD) gestational age (wks): 37.10 (0.23) vs. 37 (0.55) Preterm (< 37 wks): RR 0.93 (95% CI 0.60, 1.39) Large for gestational age (LGA): RR 1.75 (95% CI 0.92, 3.60) Shoulder dystocia: RR 0.98 (95% CI 0.33, 3.00) Admission to NICU: RR 0.91 (95% CI 0.63, 1.21) Respiratory distress: RR 0.69 (95% CI 0.40, 1.13) Need for mechanical ventilation: RR 0.47 (95% CI 0.17, 1.20) Neonatal hypoglycemia: RR 1.02 (95% CI 0.69, 1.54) Need for I/v glucose: RR 1.24 (95% CI 0.78, 2.10) Mean (SD) birth weight (gm): 3158 (660.2) vs. 2707 (812.7) APGAR < 7 (at 5 min): RR 1.13 (95% CI 0.40, 3.33) Small for gestational age (SGA): RR 0.77 (95% CI 0.32, 1.83) Neonatal hyperbilirubinemia: RR 1.20 (95% CI 0.68, 2.22)
Chico et al. (2016) [29]	Retrospective cohort	Spain	Women with singleton gestation and type 1 diabetes; Median age of 31 years; Mean pre-pregnancy BMI of 23.9 kg/m2	Glargine	1210 (356-glargine; 854- neutral protamine Hagedorn)	Maternal outcomes Symptomatic hypoglycaemia: RR 0.89 (95% CI 0.64, 1.25) Hypertensive disorder: RR 0.93 (95% CI 0.66, 1.30) Mean (SD) gestational weight gain (kg): 12 (1.63) vs. 12.5 (1.47) Caesarean section: RR 1.10 (95% CI 0.97, 1.26) Hb A1c (%) (mean, SD): 1^st^ trimester: 6.16 (0.34) vs. 6.33 (0.39) 2^nd^ trimester: 5.70 (0.24) vs. 5.79 (0.27) 3^rd^ trimester: 5.70 (0.25) vs. 5.83 (0.27) Foetal/neonatal outcomes Mean (SD) gestational age (wks): 38 (0.25) vs. 38 (0.24) Preterm (< 37 wks): RR 0.97 (95% CI 0.75, 1.24) Large for gestational age (LGA): RR 0.99 (95% CI 0.85, 1.14) Small for gestational age (SGA): RR 1.21 (95% CI 0.53, 2.81) Shoulder dystocia: RR 0.78 (95% CI 0.31, 1.94) Respiratory distress: RR 0.78 (95% CI 0.52, 1.17) Neonatal hypoglycemia: RR 1.14 (95% CI 0.89, 1.46) Mean (SD) birth weight (gm): 3495 (196.2) vs. 3495 (205.7) APGAR < 7 (at 5 min): RR 0.79 (95% CI 0.35, 1.76) Neonatal sepsis: RR 1.40 (95% CI 0.63, 1.53) Stillbirth: RR 0.37 (95% CI 0.08, 1.65) Perinatal mortality: RR 0.61 (95% CI 0.20, 1.80) Spontaneous abortion: RR 1.25 (95% CI 0.84, 1.86) Congenital malformation: RR 0.83 (95% CI 0.45, 1.55)
Bartal et al. (2020) [30]	Retrospective cohort	USA	Women with singleton gestation and type 2 diabetes; gestational diabetes mellitus (GDM) diagnosed before 20 weeks gestation; majority aged 20–34 yrs (55%); BMI ≥ 30 kg/m2 (> 75%); 80% multiparous	Insulin analogues (glargine and detemir)	233 (114-basal insulin analogues; 119- neutral protamine Hagedorn)	Maternal outcomes Symptomatic hypoglycaemia: RR 0.72 (95% CI 0.35, 1.45) Hypertensive disorder: RR 0.99 (95% CI 0.63, 1.55) Caesarean section: RR 0.93 (95% CI 0.75, 1.15) Endometritis: RR 2.09 (95% CI 0.39, 11.2) Wound infection or dehiscence: RR 0.10 (95% CI 0.02, 0.80) Hospitalization for glucose control: RR 1.26 (95% CI 0.76, 2.08) Induction of labour: RR 0.89 (95% CI 0.57, 1.37) Hospitalization during pregnancy: RR 1.37 (95% CI 0.92, 2.05) Foetal/neonatal outcomes Mean (SD) gestational age (wks): 36.2 (2.1) vs. 36.1 (2.5) Preterm (< 37 wks): RR 0.93 (95% CI 0.68, 1.26) Large for gestational age (LGA): RR 1.56 (95% CI 0.89, 2.73) Shoulder dystocia: RR 1.39 (95% CI 0.32, 6.08) Admission to NICU: RR 1.06 (95% CI 0.77, 1.46) Respiratory distress: RR 0.58 (95% CI 0.22, 1.55) Need for mechanical ventilation: RR 0.68 (95% CI 0.14, 3.24) Neonatal hypoglycemia: RR 1.30 (95% CI 0.88, 1.92) Mean (SD) birth weight (gm): 3186.1 (751.9) vs. 2993.9 (800.6) APGAR < 7 (at 5 min): RR 3.24 (95% CI 0.43, 24.53) Neonatal sepsis: RR 0.42 (95% CI 0.08, 2.11) Perinatal mortality: RR 1.89 (95% CI 0.27, 13.32) Neonatal hyperbilirubinemia: RR 0.59 (95% CI 0.34, 1.05)
Herrera et al. (2015) [31]	RCT	USA	Women with singleton gestation; type 2 diabetes (16%), remaining with type 1 diabetes (84%); in majority, GDM diagnosed after 24 weeks gestation (68%); median age of 35 years; Median BMI of 28 kg/m2	Detemir	87 (42-Detemir; 45- neutral protamine Hagedorn)	Maternal outcomes Symptomatic hypoglycaemia: RR 0.74 (95% CI 0.39, 1.40) Mean (SD) gestational weight gain (kg): 12.6 (6.9) vs. 12.9 (5.3) Foetal/neonatal outcomes Mean (SD) gestational age (wks): 38.9 (0.43) vs. 38.8 (0.25) Mean (SD) birth weight (gm): 3230 (167.5) vs. 3235 (237.5) Neonatal hypoglycemia: RR 0.21 (95% CI 0.01, 4.33) Admission to NICU: RR 0.54 (95% CI 0.14, 2.00) APGAR < 7 (at 5 min): RR 3.21 (95% CI 0.13, 76.6)
Hod et al. (2014) [32]	RCT	Multicentric	Women with singleton gestation and type 1 diabetes; mean age of 30 years; Mean BMI of 25 kg/m2	Detemir	310 (152-Detemir; 158- neutral protamine Hagedorn)	Maternal outcomes Hypertensive disorder: RR 1.49 (95% CI 0.71, 3.09) Caesarean section: RR 0.90 (95% CI 0.74, 1.11) Foetal/neonatal outcomes Mean (SD) gestational age (wks): 38.2 (1.9) vs. 37.8 (1.5) Preterm (< 37 wks): RR 0.71 (95% CI 0.40, 1.26) Large for gestational age (LGA): RR 0.74 (95% CI 0.46, 1.21) SGA: RR 3.06 (95% CI 0.32, 29.1) Neonatal hypoglycemia: RR 0.65 (95% CI 0.32, 1.30) Mean (SD) birth weight (gm): 3504 (645) vs. 3571 (601) Spontaneous abortion: RR 1.27 (95% CI 0.52, 3.14) Perinatal mortality: RR 2.04 (95% CI 0.19, 22.3) Congenital malformation: RR 1.02 (95% CI 0.39, 2.65)
Ji et al. (2020) [33]	RCT	China	Women with singleton gestation and type 1 diabetes; mean age of 31 years; Mean BMI of 25 kg/m2; mean gestational age at enrolment- 28 wks; type of diabetes (?)	Detemir	240 (120-Detemir; 120- neutral protamine Hagedorn)	Maternal outcomes Symptomatic hypoglycaemia: RR 0.50 (95% CI 0.28, 0.90) HbA1c in 3^rd^ trimester (Mean, SD): 5.66 (0.83) vs. 5.80 (0.81) Hypertensive disorder: RR 0.61 (95% CI 0.33, 1.13) Mean (SD) gestational weight gain (kg): 12.21 (3.84) vs. 11.99 (4.11) Caesarean section: RR 1.05 (95% CI 0.87, 1.28) Wound infection or dehiscence: RR 0.83 (95% CI 0.44, 1.57) Foetal/neonatal outcomes Mean (SD) gestational age (wks): 38.64 (2.19) vs. 38.15 (3.05) Preterm (< 37 wks): RR 0.83 (95% CI 0.44, 1.58) Large for gestational age (LGA): RR 1.00 (95% CI 0.43, 2.31) Admission to NICU: RR 0.92 (95% CI 0.63, 1.35) Respiratory distress: RR 1.00 (95% CI 0.21, 4.85) Neonatal hypoglycemia: RR 0.50 (95% CI 0.18, 1.42) Mean (SD) birth weight (gm): 3257.6 (496.6) vs. 3179.7 (671.8) Congenital malformation: RR 1.00 (95% CI 0.21, 4.85) Neonatal hyperbilirubinemia: RR 0.91 (95% CI 0.69, 1.21)
Mathiesen et al. (2012) [34]	RCT	Multicentric	Women with singleton gestation and type 1 diabetes; mean age of 30 years; Mean BMI of 25 kg/m2; women with type 1 diabetes	Detemir	310 (152-Detemir; 158- neutral protamine Hagedorn)	Maternal outcomes Symptomatic hypoglycaemia: RR 1.11 (95% CI 0.89, 1.38) HbA1c in 3rd trimester (Mean, SD): 6.39 (0.71) vs. 6.44 (0.68) Mean (SD) gestational weight gain (kg): 11.5 (2.5) vs. 11.0 (2.84)
Sleeman et al. (2019) [35]	Retrospective cohort	USA	Women with singleton gestation and type 2 diabetes (65%); mean age of 31 years; Mean HbA1c at enrolment of 8.0%	Insulin analogues (glargine and detemir)	63 (44-basal insulin analogues; 19- neutral protamine Hagedorn)	Maternal outcomes Symptomatic hypoglycaemia: RR 1.73 (95% CI 0.21, 14.5) HbA1c in 3rd trimester (Mean, SD): 6.3 (1.2) vs. 6.5 (1.3) Hypertensive disorder: RR 0.37 (95% CI 0.08, 1.62) Caesarean section: RR 0.96 (95% CI 0.65, 1.42) Foetal/neonatal outcomes Preterm (< 37 wks): RR 0.95 (95% CI 0.51, 1.76) Large for gestational age (LGA): RR 0.74 (95% CI 0.21, 2.55) Admission to NICU: RR 0.74 (95% CI 0.44, 1.23) Respiratory distress: RR 0.66 (95% CI 0.27, 1.64) Neonatal hypoglycemia: RR 1.72 (95% CI 0.91, 3.24) Spontaneous abortion: RR 1.84 (95% CI 0.24, 14.4) Neonatal hyperbilirubinemia: RR 0.66 (95% CI 0.27, 1.64)
Cianni et al. (2008) [36]	Retrospective cohort	Italy	Women with singleton gestation and type 1 diabetes; mean age of 30.5 years; Mean BMI at enrolment of 24 kg/m2	Glargine	101 (43-glargine; 58- neutral protamine Hagedorn)	Maternal outcomes Symptomatic hypoglycaemia: RR 0.77 (95% CI 0.24, 2.47) HbA1c in 1st trimester (Mean, SD): 6.77 (1.32) vs. 7.6 (1.09) HbA1c in 3rd trimester (Mean, SD): 6.5 (0.79) vs. 6.5 (0.91) Hypertensive disorder: RR 0.67 (95% CI 0.06, 7.2) Caesarean section: RR 0.99 (95% CI 0.81, 1.22) Mean (SD) gestational weight gain (kg): 14.1 (4.1) vs. 13.3 (4.4) Foetal/neonatal outcomes Large for gestational age (LGA): RR 1.07 (95% CI 0.68, 1.68) Admission to NICU: RR 1.14 (95% CI 0.57, 2.30) Neonatal hypoglycemia: RR 0.81 (95% CI 0.32, 2.05) Congenital malformation: RR 0.87 (95% CI 0.15, 4.97) Neonatal hyperbilirubinemia: RR 0.93 (95% CI 0.44, 1.98)
Egerman et al. (2009) [37]	Retrospective cohort	USA	Women with singleton gestation; type of diabetes not reported; mean age of 29 years; Mean BMI at enrolment of > 30 kg/m2; mean gestational age at enrolment (19 weeks); majority were multigravida	Glargine	114 (65-glargine; 49- neutral protamine Hagedorn)	Maternal outcomes Hypertensive disorder: RR 2.01 (95% CI 0.85, 4.76) HbA1c in 3rd trimester (Mean, SD): 7.04 (1.4) vs. 7.48 (2.09) Caesarean section: RR 1.08 (95% CI 0.84, 1.40) Mean (SD) gestational weight gain (kg): 13.06 (9.1) vs. 13.69 (10.6) Foetal/neonatal outcomes Admission to NICU: RR 0.98 (95% CI 0.93, 1.05) Neonatal hypoglycemia: RR 1.19 (95% CI 0.64, 2.22) Congenital malformation: RR 1.08 (95% CI 0.44, 2.63) Respiratory distress: RR 0.75 (95% CI 0.23, 2.46) Shoulder dystocia: RR 0.08 (95% CI 0.01, 1.53) Mean (SD) gestational age (wks): 37.3 (2.8) vs. 37.7 (3.3) Mean (SD) birth weight (gm): 3415 (1231) vs. 3351 (1083) Neonatal hyperbilirubinemia: RR 1.68 (95% CI 0.98, 2.88)
Fang et al. (2009) [38]	Retrospective cohort	USA	Women with singleton gestation; type of diabetes not reported; mean age of 30 years; majority were multigravida	Glargine	112 (52-glargine; 60- neutral protamine Hagedorn)	Maternal outcomes Symptomatic hypoglycaemia: RR 3.45 (95% CI 0.14, 82.9) Hypertensive disorder: RR 0.29 (95% CI 0.03, 2.50) Mean (SD) gestational weight gain (kg): 10.8 (8.1) vs. 11.6 (7.7) Caesarean section: RR 0.87 (95% CI 0.63, 1.20) HbA1c (%), third trimester: 6.55 (0.30) vs. 6.70 (0.21) Foetal/neonatal outcomes Mean (SD) gestational age (wks): 38 (1.0) vs. 37.5 (1.2) Preterm (< 37 wks): RR 0.62 (95% CI 0.27, 1.44) Large for gestational age (LGA): RR 0.77 (95% CI 0.38, 1.56) Admission to NICU: RR 0.32 (95% CI 0.09, 1.07) Respiratory distress: RR 3.45 (95% CI 0.14, 82.9) Neonatal hypoglycemia: RR 0.19 (95% CI 0.02, 1.55) Mean (SD) birth weight (gm): 3501.3 (473.1) vs. 3296.4 (525.6) APGAR < 7 (at 5 min): RR 0.38 (95% CI 0.04, 3.59) Neonatal hyperbilirubinemia: RR 0.48 (95% CI 0.18, 1.27)
Imbergamo et al. (2008) [39]	Case–control	Italy	Women with singleton gestation; type 1 diabetes; mean age of around 27.5 years	Glargine	30 (15-glargine; 15- neutral protamine Hagedorn)	Maternal outcomes Symptomatic hypoglycaemia: RR 0.71 (95% CI 0.29, 1.75) Hypertensive disorder: RR 1.25 (95% CI 0.41, 3.77) Mean (SD) gestational weight gain (kg): 12.4 (3.8) vs. 13.5 (3.9) Caesarean section: RR 1.00 (95% CI 0.60, 1.66) HbA1c (%), first trimester: 6.86 (0.95) vs. 7.79 (1.13) HbA1c (%), second trimester: 6.16 (0.67) vs. 6.62 (0.80) HbA1c (%), third trimester: 6.23 (0.86) vs. 6.47 (0.95) Foetal/neonatal outcomes Mean (SD) gestational age (wks): 36.42 (2.21) vs. 37.04 (1.27) Large for gestational age (LGA): RR 1.75 (95% CI 0.64, 4.74) Respiratory distress: RR 7.00 (95% CI 0.39, 124.8) Neonatal hypoglycemia: RR 4.00 (95% CI 0.50, 31.7) Mean (SD) birth weight (gm): 3278 (756) vs. 3503 (455) APGAR < 7 (at 5 min): RR 9.00 (95% CI 0.53, 153.8) Neonatal hyperbilirubinemia: RR 5.00 (95% CI 0.26, 96.1)
Negrato et al. (2010) [40]	Prospective cohort	Brazil	Women with singleton gestation; gestational diabetes mellitus (GDM) diagnosed between 24–28 weeks gestation; mean age of 30 years; women with both pregestational (n = 56) and gestational diabetes (n = 82); type of diabetes not specified	Glargine	138 (55-glargine; 83- neutral protamine Hagedorn)	Maternal outcomes Symptomatic hypoglycaemia: RR 0.12 (95% CI 0.01, 2.00) Hypertensive disorder: RR 0.28 (95% CI 0.09, 0.93) Mean (SD) gestational weight gain (kg): 11.2 (9.4) vs. 10.6 (6.8) Caesarean section: RR 0.99 (95% CI 0.92, 1.08) HbA1c (%), third trimester: 6.1 (0.8) vs. 6.1 (0.8) Foetal/neonatal outcomes Mean (SD) gestational age (wks): 37.7 (1.43) vs. 36.6 (4.85) Large for gestational age (LGA): RR 1.04 (95% CI 0.68, 1.58) Small for gestational age (SGA): RR 0.30 (95% CI 0.02, 6.13) Respiratory distress: RR 1.26 (95% CI 0.40, 3.92) Neonatal hypoglycemia: RR 0.57 (95% CI 0.16, 2.04) Mean (SD) birth weight (gm): 3450 (640) vs. 3300 (520) APGAR < 7 (at 5 min): RR 0.50 (95% CI 0.02, 12.06) Neonatal hyperbilirubinemia: RR 0.30 (95% CI 0.04, 2.51) Preterm (< 37 wks): RR 0.78 (95% CI 0.46, 1.32) Admission to NICU: RR 0.41 (95% CI 0.12, 1.41) Congenital malformation: RR 0.30 (95% CI 0.07, 1.33) Perinatal mortality: RR 0.30 (95% CI 0.02, 6.13)
Poyhonen-Alho et al. (2007) [41]	Retrospective chart review	Finland	Pregnant women with singleton pregnancy and type 1 diabetes; women in both groups did not differ in age, parity, age at diagnosis or duration of diabetes, or occurrence of retinopathy or nephropathy before pregnancy	Glargine	91 (42-glargine; 49- neutral protamine Hagedorn)	Maternal outcomes Symptomatic hypoglycaemia: RR 2.33 (95% CI 0.45, 12.1) Hypertensive disorder: RR 0.58 (95% CI 0.19, 1.80) Caesarean section: RR 1.03 (95% CI 0.69, 1.53) HbA1c (%), first trimester: 7.6 (0.2) vs. 7.2 (0.3) HbA1c (%), second trimester: 6.7 (0.1) vs. 6.8 (0.3) HbA1c (%), third trimester: 6.9 (0.1) vs. 6.9 (0.2) Foetal/neonatal outcomes Mean (SD) gestational age (wks): 36.0 (0.85) vs. 37.0 (0.50) Respiratory distress: RR 2.04 (95% CI 0.64, 6.49) Neonatal hypoglycemia: RR 0.82 (95% CI 0.45, 1.52) Mean (SD) birth weight (gm): 3796 (101) vs. 3789 (68) Neonatal hyperbilirubinemia: RR 0.89 (95% CI 0.49, 1.61) Preterm: RR 2.33 (95% CI 0.22, 24.8) Congenital malformation: RR 3.49 (95% CI 0.15, 83.4) Perinatal mortality: RR 1.17 (95% CI 0.08, 18.1) Shoulder dystocia: RR 0.39 (95% CI 0.04, 3.60) Neonatal sepsis: RR 1.16 (95% CI 0.17, 7.93)
Price et al. (2007) [42]	Case-control	UK	Women with singleton gestation with type 1 diabetes; mean age of around 32 years; Majority were pre-obese or obese (BMI > 25 kg/m2; majority were multigravida	Glargine	64 (32-glargine; 32- neutral protamine Hagedorn)	Maternal outcomes HbA1c (%), third trimester: 6.2 (0.85) vs. 6.1 (0.60) Foetal/neonatal outcomes Mean (SD) gestational age (wks): 38.1 (0.47) vs. 38.1 (0.52) Large for gestational age (LGA): RR 0.92 (95% CI 0.50, 1.70) Respiratory distress: RR 7.00 (95% CI 0.38, 130.3) Neonatal hypoglycemia: RR 1.33 (95% CI 0.65, 2.72) Mean (SD) birth weight (gm): 3537 (520) vs. 3594 (429) Congenital malformation: RR 0.67 (95% CI 0.12, 3.73) Admission to NICU: RR 1.40 (95% CI 0.50, 3.95)
Smith et al. (2009) [43]	Retrospective chart review	USA	Women with singleton gestation; both type 1 and type 2 diabetes; age range of 25–35 years; majority were multigravida	Glargine	52 (27-glargine; 25- neutral protamine Hagedorn)	Maternal outcomes Hypertensive disorder: RR 0.37 (95% CI 0.08, 1.74) Caesarean section: RR 1.21 (95% CI 0.75, 1.95) Foetal/neonatal outcomes Mean (SD) gestational age (wks): 36.3 (0.96) vs. 37.0 (0.7) Mean (SD) birth weight (gm): 3294 (189) vs. 3274 (137) Admission to NICU: RR 1.08 (95% CI 0.42, 2.78) APGAR < 7 (at 5 min): RR 2.78 (95% CI 0.12, 65.4)
Imbergamo et al. (2012) [44]	Case-control	Italy	Women with singleton gestation; type 1 diabetes; mean age of around 29 years	Detemir	16 (8-detemir; 8- neutral protamine Hagedorn)	Maternal outcomes HbA1c (%), first trimester: 7.38 (0.99) vs. 7.60 (1.33) HbA1c (%), second trimester: 6.58 (0.66) vs. 6.43 (0.77) HbA1c (%), third trimester: 6.77 (1.24) vs. 6.32 (1.15) Mean (SD) gestational weight gain (kg): 14 (3.8) vs. 14.46 (4.5) Symptomatic hypoglycaemia: RR 3.00 (95% CI 0.39, 23.1) Hypertensive disorder: RR 0.14 (95% CI 0.01, 2.38) Foetal/neonatal outcomes Mean (SD) gestational age (wks): 36.50 (0.92) vs. 37.35 (1.55) Large for gestational age (LGA): RR 0.50 (95% CI 0.06, 4.47) Mean (SD) birth weight (gm): 3326 (401) vs. 3489 (572) Neonatal hyperbilirubinemia: RR 5.00 (95% CI 0.28, 90.2)

### Quality assessment of included studies

Findings of the quality assessment have been presented in supplementary document (Additional file 1: Figures S1, S2 and Table S2). For the RCTs, the bias arising from the randomization process was judged to be present only in one out of the five studies. Similarly, bias due to deviations from intended interventions was judged to be present only in one RCT. In two of the five studies, there were concerns regarding bias in the measurement of the outcomes. None of the RCTs had missing outcome data or bias in the selection of the reported result. For the observational studies, all the studies had a NOS score of either eight or nine (out of the maximum attainable score of nine). Overall, the assessments indicate most of the included studies to be of fairly good quality.

### Effect on maternal outcomes

**Fig. 2:**
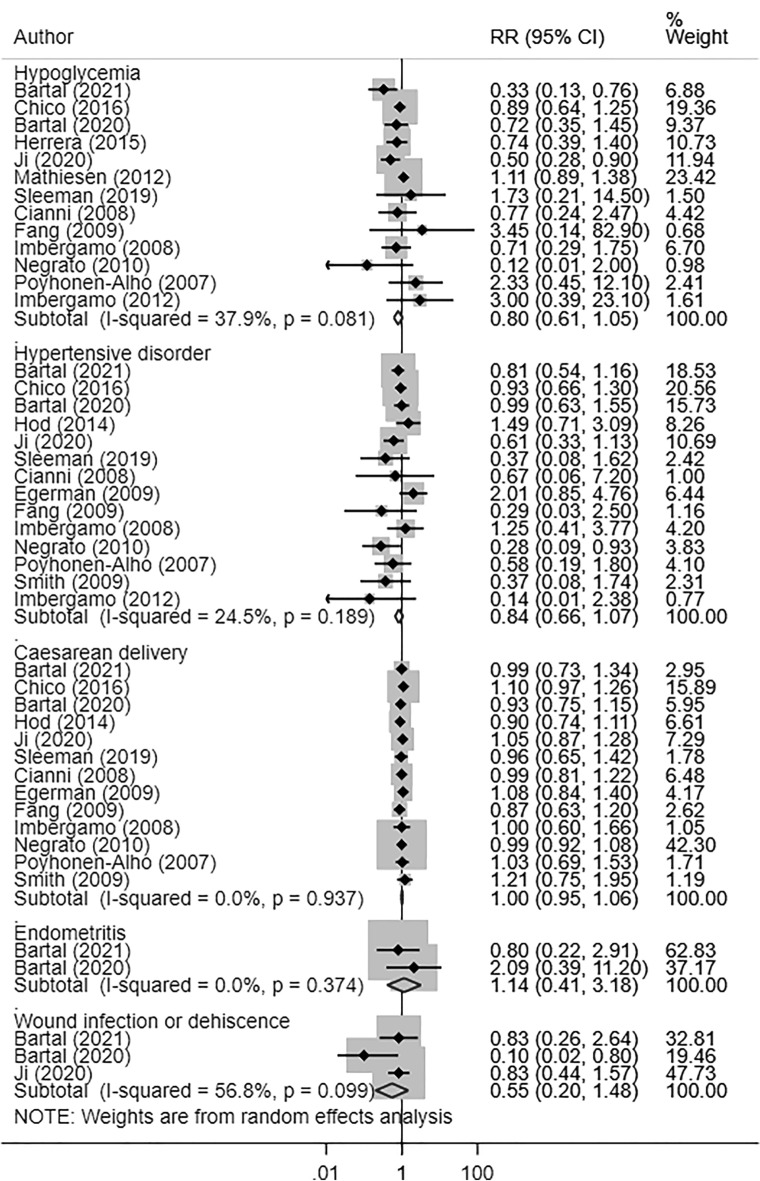
Maternal outcomes in women with gestational diabetes receiving long-acting insulin analogues (glargine and/or detemir), compared to intermediate acting neutral protamine Hagedron

The risk of maternal hypoglycaemia (RR 0.80, 95% CI 0.61, 1.05; N = 13, I^2^ = 37.9%), hypertensive disorder (RR 0.84, 95% CI 0.66, 1.07; N = 14, I^2^ = 24.5%), caesarean delivery (RR 1.00, 95% CI 0.95, 1.06; N = 13, I^2^ = 0.0%), endometritis (RR 1.14, 95% CI 0.41, 3.18; N = 2, I^2^ = 0.0%) and wound infection or dehiscence (RR 0.55, 95% CI 0.20, 1.48; N = 3, I^2^ = 56.8%) were similar among pregnant women with GDM managed using either long-acting insulin analogues or NPH (Fig. 2). There was no evidence of publication bias (P = 0.22 for maternal hypoglycaemia; P = 0.75 for hypertensive disorder; P = 0.89 for caesarean delivery; P = 0.47 for endometritis and P = 0.19 for wound infection or dehiscence). For some of these maternal outcomes, the funnel plots for visually assessing publication bias have been presented in supplementary document (Additional file 1: Figures S3–S5).

**Fig. 3:**
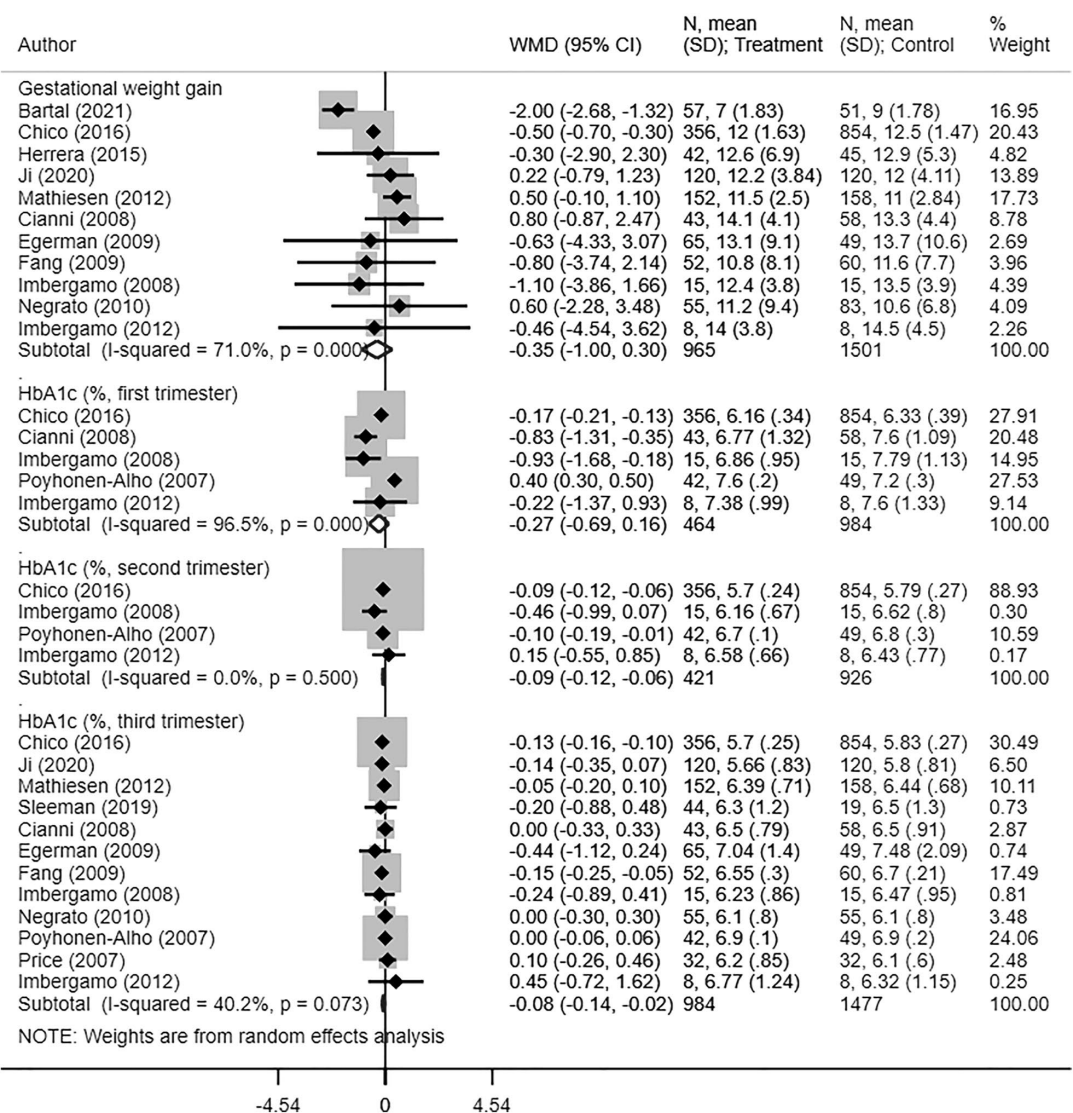
Maternal gestational weight gain (Kg) and glycosylated haemoglobin (HbA1c, %) in women with gestational diabetes receiving long-acting insulin analogues (glargine and/or detemir), compared to intermediate acting neutral protamine Hagedron

Women that received long-acting insulin analogues had similar gestational weight gain (Kg) as those receiving NPH (WMD −0.35, 95% CI −1.00, 0.30; N = 11, I^2^ = 71.0%) (Fig. 3). Glycosylated haemoglobin (HbA1c, %) values for the first trimester were similar among both groups of women (WMD −0.27, 95% CI −0.69, 0.16; N = 5, I^2^ = 96.5%). However, those receiving long-acting insulin analogues had significantly lower values in the second (WMD −0.09, 95% CI −0.12, −0.06; N = 4, I^2^ = 0.0%) and third trimesters (WMD − 0.08, 95% CI −0.14, −0.02; N = 12, I^2^ = 40.2%) (Fig. 3). For all the maternal outcomes considered, the quality of pooled estimates obtained was judged to be “Very Low” according to the GRADE assessment criteria (Additional file 1: Table S3).

Subgroup analysis based on the type of long-acting insulin analogue (i.e., glargine or detemir) indicated no significant differences in the majority of the maternal outcomes when compared to NPH (Table 2, Additional file 1: Figures S6–S9). However, gestational weight gain (kg) (WMD − 0.48, 95% CI −0.68, −0.29; N = 6, I^2^ = 0.0%), HbA1c (%) in second (WMD − 0.09, 95% CI −0.12, −0.06; N = 3, I^2^ = 0.0%) and third trimesters (WMD − 0.08, 95% CI −0.16, −0.01; N = 8, I^2^ = 58.5%) were significantly lower in those managed using glargine (Table 2, Additional file 1: figure S7).

**Table 2:** Maternal outcomes based on the type of long-acting insulin analogue used, compared to intermediate acting insulin (neutral protamine Hagedron, NPH)

Maternal outcomes	Glargine	Detemir
	Pooled effect size (95% CI); (N = total number of studies; I^2^)	
Hypoglycemia	RR 0.88 (0.65, 1.18); (N = 6; I^2^ = 0.0%)	RR 0.72 (0.42, 1.23); (N = 5; I^2^ = 71.2%)
Hypertensive disorder	RR 0.88 (0.67, 1.17); (N = 8; I^2^ = 33.2%)	RR 0.82 (0.52, 1.29); (N = 4; I^2^ = 40.3%)
Caesarean delivery	RR 1.02 (0.96, 1.08); (N = 8; I^2^ = 0.0%)	RR 0.98 (0.86, 1.11); (N = 3; I^2^ = 0.0%)
Endometritis	**––**	RR 0.80 (0.22, 2.91); (N = 1)
Wound infection/dehiscence	**––**	RR 0.83 (0.48, 1.45); (N = 2; I^2^ = 0.0%)
Gestational weight gain (kg)	WMD −0.48 (−0.68, −0.29)**;** (N = 6; I^2^ = 0.0%) *	WMD −0.42 (−1.81, 0.97)**;** (N = 5; I^2^ = 87.3%)
HbA1c (%, 1st trimester)	WMD −0.27 (−0.72, 0.17)**;** (N = 4; I^2^ = 97.3%)	WMD − 0.22 (−1.37, 0.93)**;** (N = 1)
HbA1c (%, 2nd trimester)	WMD −0.09 (−0.12, −0.06)**;** (N = 3; I^2^ = 0.0%) *	WMD 0.15 (−0.55, 0.85)**;** (N = 1)
HbA1c (%, 3rd trimester)	WMD −0.08 (−0.16, −0.01)**;** (N = 8; I^2^ = 58.5%) *	WMD − 0.08 (−0.20, 0.05)**;** (N = 3; I^2^ = 0.0%)

*RR* relative risk, *WMD* weighted mean difference

^*^statistically significant at P < 0.05

### Effect on perinatal and neonatal outcomes

**Fig. 4:**
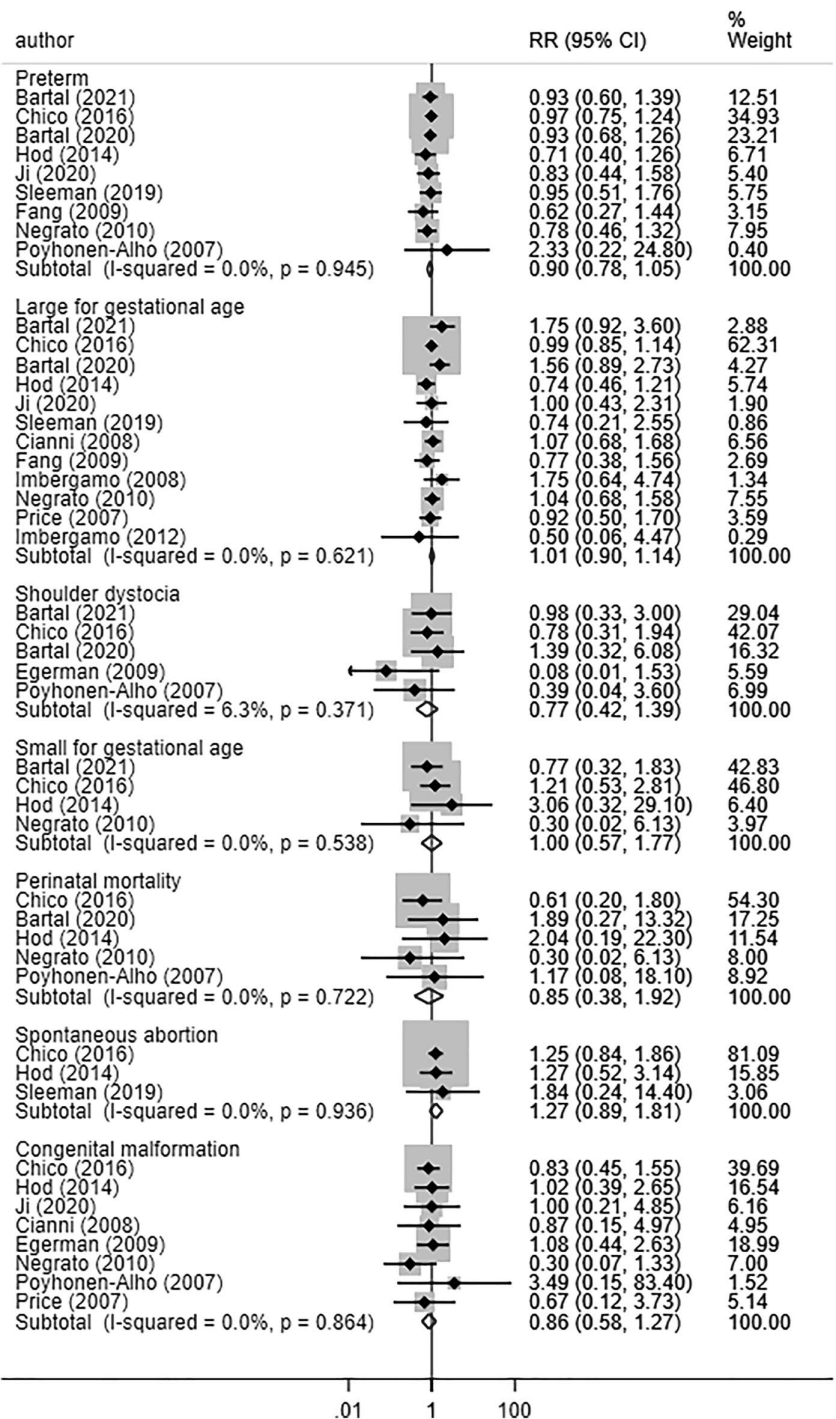
Birth outcomes in women with gestational diabetes receiving long-acting insulin analogues (glargine and/or detemir), compared to intermediate acting neutral protamine Hagedron

The risk of preterm birth (RR 0.90, 95% CI 0.78, 1.05; N = 9, I^2^ = 0.0%), large for gestational age (LGA) (RR 1.01, 95% CI 0.90, 1.14; N = 12, I^2^ = 0.0%), small for gestational age (SGA) (RR 1.00, 95% CI 0.57, 1.77; N = 4, I^2^ = 0.0%) and congenital abnormalities (RR 0.86, 95% CI 0.58, 1.27; N = 8, I^2^ = 0.0%) was similar between babies born to women managed using long-acting insulin analogues or NPH (Fig. 4). Similarly, the risk of spontaneous abortion (RR 1.27, 95% CI 0.89, 1.81; N = 3, I^2^ = 0.0%), perinatal mortality (RR 0.85, 95% CI 0.38, 1.92; N = 5, I^2^ = 0.0%), and shoulder dystocia (RR 0.77, 95% CI 0.42, 1.39; N = 5, I^2^ = 6.3%) was similar in the two groups (Fig. 4). There was no evidence of publication bias (P = 0.23 for prematurity; P = 0.61 for LGA; P = 0.48 for SGA; P = 0.44 for shoulder dystocia; P = 0.17 for perinatal mortality; P = 0.25 for spontaneous abortion and P = 0.11 for congenital abnormalities). The funnel plots for visually assessing publication bias have been presented in supplementary document (Additional file 1: Figures S10-S14).

**Fig. 5:**
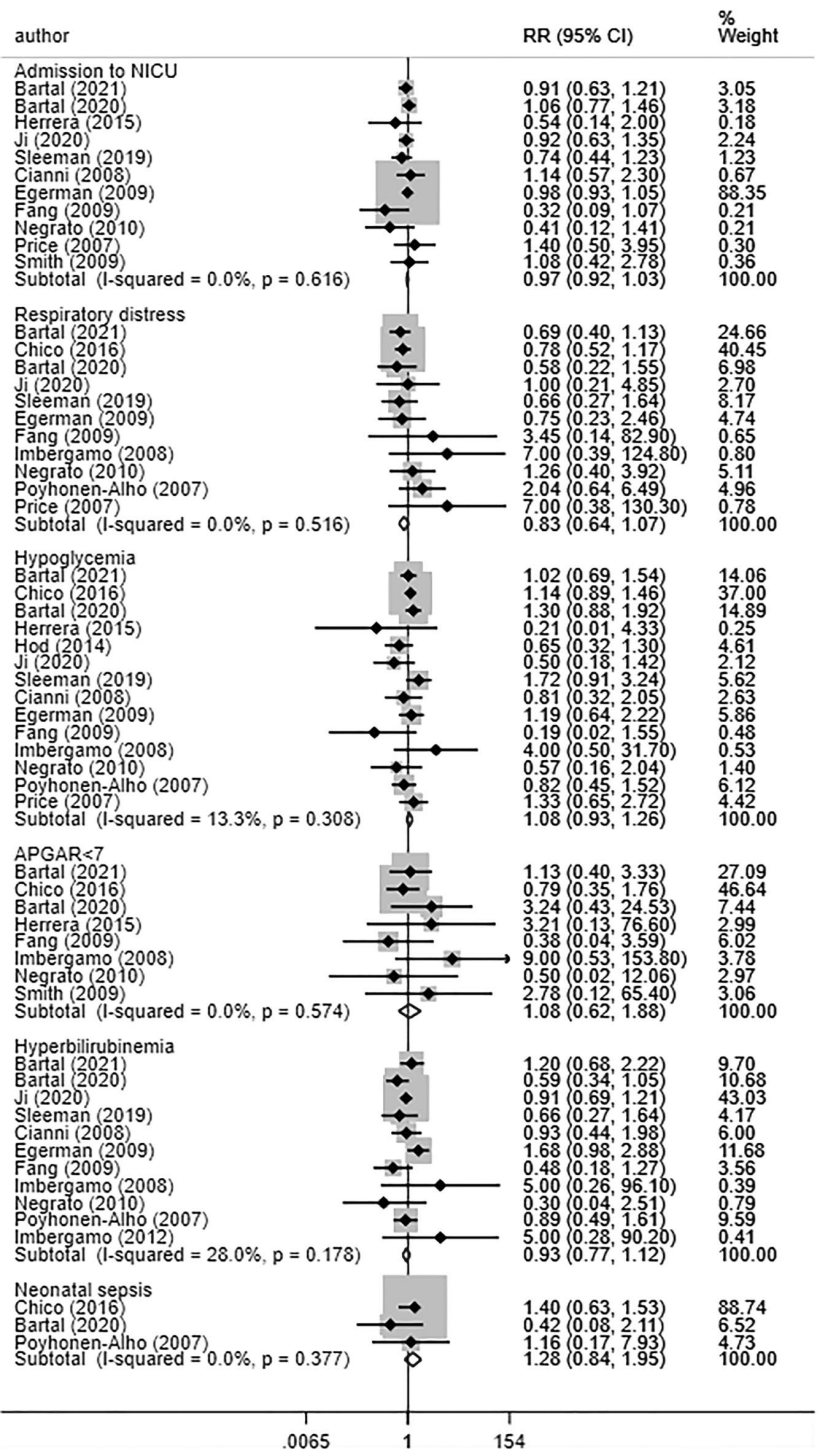
Neonatal morbidity and associated clinical outcomes in women with gestational diabetes receiving long-acting insulin analogues (glargine and/or detemir), compared to intermediate acting neutral protamine Hagedron

**Fig. 6:**
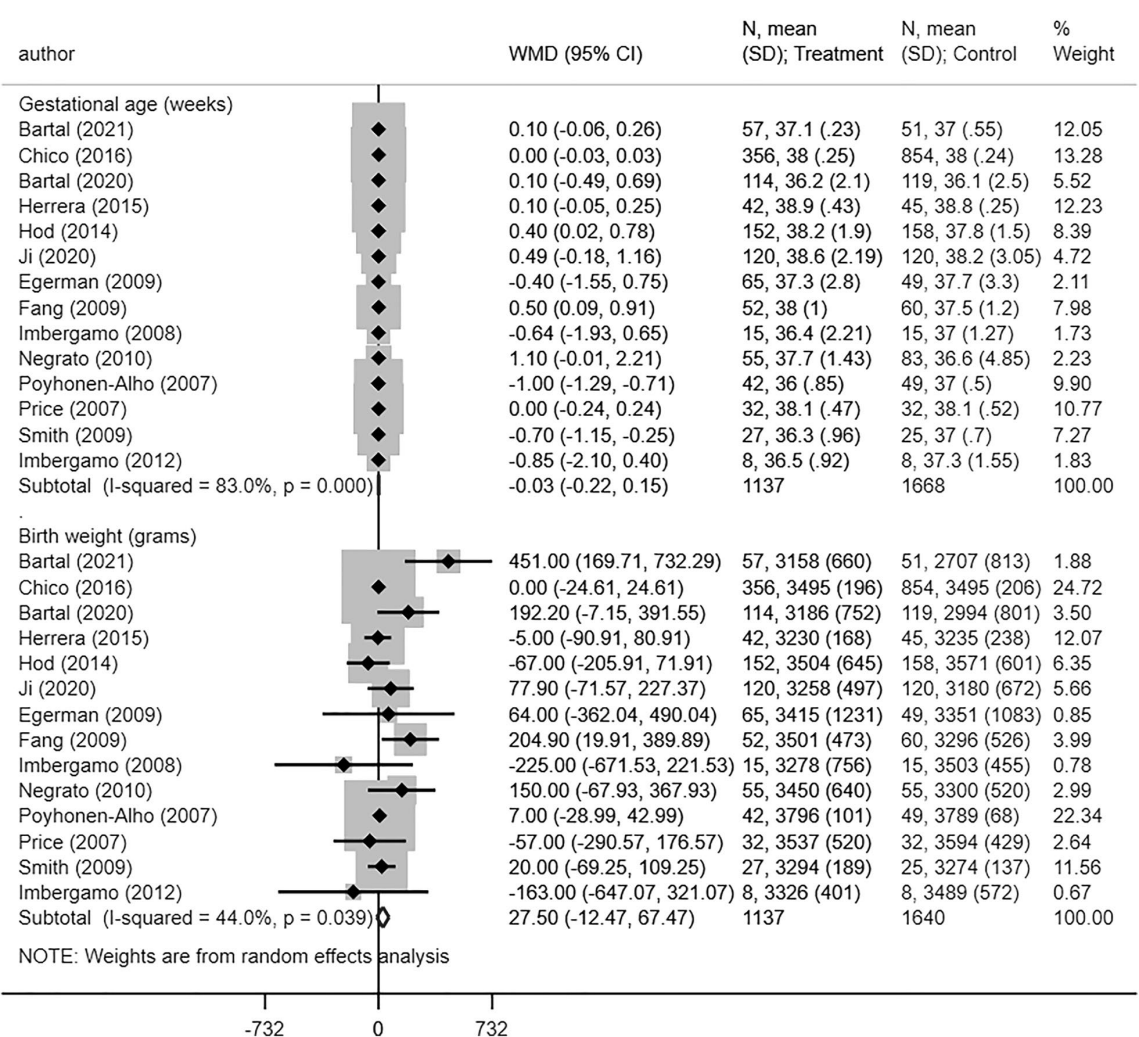
Mean gestational age and birth weight in neonates born to mothers with gestational diabetes receiving long-acting insulin analogues (glargine and/or detemir), compared to intermediate acting neutral protamine Hagedron

No statistically significant differences were found for risk of admission to neonatal intensive care unit (NICU) (RR 0.97, 95% CI 0.92, 1.03; N = 11, I^2^ = 0.0%), respiratory distress (RR 0.83, 95% CI 0.64, 1.07; N = 11, I^2^ = 0.0%), neonatal hypoglycaemia (RR 1.08, 95% CI 0.93, 1.26; N = 14, I^2^ = 13.3%), 5 min APGAR score < 7 (RR 1.08, 95% CI 0.62, 1.88; N = 8, I^2^ = 0.0%), neonatal hyperbilirubinemia (RR 0.93, 95% CI 0.77, 1.12; N = 11, I^2^ = 28.0%), or sepsis (RR 1.28, 95% CI 0.84, 1.95; N = 3, I^2^ = 0.0%) between babies born to women managed by long-acting insulin analogues or NPH (Fig. 5). Mean gestational age (in weeks) (WMD − 0.03, 95% CI − 0.22, 0.15; N = 14, I^2^ = 83.0%) and mean birth weight (WMD 27.50, 95% CI − 12.47, 67.47; N = 14, I^2^ = 44.0%) were similar in both groups (Fig. 6). We did not find any evidence of publication bias using the Egger’s test (P = 0.25 for admission to NICU; P = 0.93 for respiratory distress; P = 0.29 for hypoglycaemia; P = 0.34 for APGAR score; P = 0.42 for hyperbilirubinemia and P = 0.31 for neonatal sepsis). For some of the outcomes, the funnel plots for visually assessing publication bias have been presented in supplementary document (Additional file 1: Figures S15–S18). For all the neonatal outcomes considered, the quality of pooled estimates obtained was judged to be “Very Low” according to the GRADE assessment criteria (Additional file 1: Table S2). Subgroup analysis based on the type of long-acting insulin analogue (i.e., glargine or detemir) indicated no significant differences in perinatal and neonatal outcomes in comparison to NPH (Table 3; Additional file 1: Figures S19–S24).

**Table 3:** Neonatal outcomes based on the type of long-acting insulin analogue used, compared to intermediate acting insulin (neutral protamine Hagedron, NPH)

Neonatal outcomes	Glargine	Detemir
	Pooled effect size (95% CI); (N = total number of studies; I^2^)	
Preterm	RR 0.91 (0.73, 1.14); (N = 4; I^2^ = 0.0%)	RR 0.84 (0.62, 1.14); (N = 3; I^2^ = 0.0%)
Large for gestational age	RR 1.00 (0.88, 1.13); (N = 6; I^2^ = 0.0%)	RR 0.97 (0.68, 1.38); (N = 4; I^2^ = 32.5%)
Shoulder dystocia	RR 0.57 (0.25, 1.26); (N = 3; I^2^ = 31.0%)	RR 0.98 (0.33, 2.95); (N = 1)
Small for gestational age	RR 1.08 (0.49, 2.42); (N = 2; I^2^ = 0.0%)	RR 0.92 (0.41, 2.08); (N = 2; I^2^ = 20.1%)
Perinatal mortality	RR 0.61 (0.23, 1.59); (N = 3; I^2^ = 0.0%)	RR 2.04 (0.19, 22.1); (N = 1)
Spontaneous abortion	RR 1.25 (0.84, 1.86); (N = 1)	RR 1.27 (0.52, 3.12); (N = 1)
Congenital malformation	RR 0.82 (0.53, 1.28); (N = 6; I^2^ = 0.0%)	RR 1.01 (0.45, 2.30); (N = 2; I^2^ = 0.0%)
Admission to NICU	RR 0.98 (0.92, 1.04); (N = 6; I^2^ = 12.9%)	RR 0.90 (0.70, 1.15); (N = 3; I^2^ = 0.0%)
Respiratory distress	RR 0.95 (0.68, 1.34); (N = 7; I^2^ = 17.1%)	RR 0.72 (0.44, 1.17); (N = 2; I^2^ = 0.0%)
Hypoglycaemia	RR 1.08 (0.89, 1.32); (N = 8; I^2^ = 0.0%)	RR 0.84 (0.61, 1.17); (N = 4; I^2^ = 5.7%)
5-min APGAR (< 7)	RR 0.89 (0.44, 1.78); (N = 5; I^2^ = 0.0%)	RR 1.25 (0.46, 3.43); (N = 2; I^2^ = 0.0%)
Hyperbilirubinemia	RR 1.05 (0.76, 1.45); (N = 6; I^2^ = 39.5%)	RR 0.97 (0.75, 1.25); (N = 3; I^2^ = 0.0%)
Neonatal sepsis	RR 1.39 (0.90, 2.14); (N = 2; I^2^ = 0.0%)	––
Gestational age (weeks)	WMD −0.18 (−0.54, 0.18); (N = 8; I^2^ = 89.1%)	WMD 0.14 (−0.01, 0.29); (N = 5; I^2^ = 29.4%)
Birth weight (grams)	WMD 8.54 (−16.9, 33.9); (N = 8; I^2^ = 10.5%)	WMD 51.9 (−81.6, 185.6); (N = 5; I^2^ = 66.6%)

*RR* relative risk, *WMD* weighted mean difference

## Discussion

Through pooling of findings from 17 studies, we found no significant differences in outcomes between those managed by long-acting analogues compared to intermediate acting NPH except for lower glycosylated haemoglobin (HbA1c, %) values in the second and third trimester among those managed by long-acting analogues. In the subgroup analysis based on the type of long-acting insulin analogue (i.e., glargine or detemir), no significant differences in outcomes were noted in comparison to NPH.

Lepercq et al. conducted a review by including eight observational studies and found no significant differences in maternal and neonatal outcomes with use of glargine compared to NPH insulin [18]. The review also noted no difference in the glycosylated haemoglobin in first and third trimester between pregnant women treated with glargine and NPH. Another review by ShiShi et al. concluded glargine and detemir to be safe treatment options for diabetes during pregnancy and noted that these long-acting insulin analogues do not increase maternal and/or neonatal complications [20]. A review by Pollex et al. noted an increase in the risk of adverse foetal outcomes with use of glargine, compared to NPH insulin. However, no significant difference in the gestational age or birth weight among babies born to mothers managed by glargine compared to NPH insulin was reported [19].

More recently, a review by Bugazia et al., found a lower risk of maternal hypoglycaemia and an increase in gestational age in mothers receiving detemir, when compared to those receiving NPH [45]. This review included 5 RCTs comparing maternal and neonatal outcomes between detemir and NPH. There were no differences in other maternal and neonatal outcomes (such as preterm birth, hypoglycaemia, birth weight and congenital anomalies). We also found similar increase in gestational age (in weeks) (WMD 0.14), which tended towards statistical significance, when only studies comparing detemir with NPH were pooled (Table 3). For the maternal hypoglycaemia, we had pooled a total of 5 studies, of which four were RCTs and one was observational). When the analysis was run with only four RCTs, we obtained an effect size similar to the one reported by Bugazia et al., denoting reduction in the risk with use of detemir. When compared to this recent review by Bugazia et al., our study offers the most comprehensive pooled evidence as it takes into account not only the 5 RCTs but observational studies also. While some may argue on the use of observational studies and its associated biases, we feel that all available data must be carefully brought into use when generating contemporary evidence and thereby, attempting to re-fashion existing clinical guidelines.

The concern with use of glargine has been that it might cross the placental barrier and by virtue of its affinity for insulin like growth factor (IGF-1) and increased mitogenic activity, compared to human insulin, can increase the risk of having a large for gestational age (LGA) baby or may lead to foetal abnormalities [46, 47]. Another potential concern with use of glargine has been the findings from in vitro studies that show its ability to stimulate DNA synthesis in human osteosarcoma cell lines, much more than human insulin [46]. However, this increased mitogenic activity has been shown to be similar to that of human insulin in other cell lines such as skeletal muscle cells, endothelium cells and normal epithelial breast cells [48–50]. Particularly, recent in vivo studies have indicated that glargine is metabolized into two active metabolites that account for the major effect of glargine and have affinity for insulin like growth factor similar to human insulin [51]. Using human placental perfusion models, studies have recently aimed at identifying the rate and extent to which glargine crosses the placental barrier at therapeutic as well as high concentrations [52, 53]. These experiments have noted negligible transplacental passage of glargine in the foetal circuit. These data seem to suggest that at therapeutic concentrations, glargine is unlikely to cross the placental barrier and effect the growing foetus. This likely explains the reason for lack of any significant effect of insulin glargine on neonatal outcomes, particularly with respect to LGA and congenital abnormalities.

Some limitations of this meta-analysis are important to consider while interpreting the findings. Included studies were observational in design and therefore, there exists a possibility that few of the important confounders are not adjusted in the statistical models considered by these studies. Also, there could be an issue of missing information in studies that retrospectively analysed medical records. Further, it is well known that high body mass index (BMI) is associated with adverse pregnancy outcomes and often co-exists with diabetes [54, 55]. It was unclear in many of the included studies that whether maternal pre-pregnancy BMI was adjusted in the analysis. Additionally, certain baseline characteristics such as socioeconomic status, ethnicity, maternal education could also impact pregnancy outcomes and most of the studies did not discuss baseline differences in these characteristics [56–58]. We did not conduct a subgroup analysis based on the type of study design (RCT and observational) as the overall pooled analysis did not indicate that such an analysis will yield any additional significant or otherwise useful finding.

## Conclusion

The present findings build upon and support the findings of previously published systematic reviews on this subject. The review noted that use of long-acting insulin analogues (glargine and detemir) led to slightly better glycaemic control in second and third trimester, as reflected by HbA1c values. However, this improved glycaemic control had no impact on maternal, perinatal and neonatal outcomes. There are significant clinical implications of these findings for use of long-acting insulin analogues in management of gestational diabetes mellitus. Clinicians have now more options available with them to manage GDM without the fear of adversely impacting maternal, perinatal or neonatal outcomes. With majority of the studies in the current meta-analysis being observational in design and the obtained quality of pooled estimates judged to be “very low” as per the GRADE criteria, the need for more randomized controlled trials to provide conclusive evidence on this subject is definitely felt.

## Supplementary Information

**Additional file 1: Table S1.** Search strategy for identification of studies to be included in the review. **Table S2.** Certainty of pooled estimates assessed using GRADE criteria. **Table S3.** Author’s judgements about study quality using the adapted Ottawa-Newcastle Risk of Bias Assessment tool. **Figure S1.** Risk of bias summary: review authors’ judgements about each risk of bias item for each included randomised controlled study. **Figure S2.** Risk of bias graph: review authors’ judgements about each risk of bias item presented as percentages across all included randomised controlled studies. **Figure S3.** Funnel plot for maternal hypoglycaemia as an outcome of interest in women with gestational diabetes receiving long-acting insulin analogues (glargine and/or detemir), compared to intermediate acting neutral protamine Hagedron. **Figure S4.** Funnel plot for caesarean delivery as an outcome of interest in women with gestational diabetes receiving long-acting insulin analogues (glargine and/or detemir), compared to intermediate acting neutral protamine Hagedron. **Figure S5.** Funnel plot for hypertensive disorder as an outcome of interest in women with gestational diabetes receiving long-acting insulin analogues (glargine and/or detemir), compared to intermediate acting neutral protamine Hagedron. **Figure S6.** Maternal outcomes in women with gestational diabetes receiving glargine, compared to intermediate acting neutral protamine Hagedron. **Figure S7.** Maternal gestational weight gain (Kg) and glycosylated haemoglobin (HbA1c, %) in women with gestational diabetes receiving glargine, compared to intermediate acting neutral protamine Hagedron. **Figure S8.** Maternal outcomes in women with gestational diabetes receiving detemir, compared to intermediate acting neutral protamine Hagedron. **Figure S9.** Maternal gestational weight gain (Kg) and glycosylated haemoglobin (HbA1c, %) in women with gestational diabetes receiving detemir, compared to intermediate acting neutral protamine Hagedron. **Figure S10.** Funnel plot for preterm birth as an outcome of interest in women with gestational diabetes receiving long-acting insulin analogues (glargine and/or detemir), compared to intermediate acting neutral protamine Hagedron. **Figure S11.** Funnel plot for large for gestational age as an outcome of interest in women with gestational diabetes receiving long-acting insulin analogues (glargine and/or detemir), compared to intermediate acting neutral protamine Hagedron. **Figure S12.** Funnel plot for small for gestational age as an outcome of interest in women with gestational diabetes receiving long-acting insulin analogues (glargine and/or detemir), compared to intermediate acting neutral protamine Hagedron. **Figure S13.** Funnel plot for congenital malformation as an outcome of interest in women with gestational diabetes receiving long-acting insulin analogues (glargine and/or detemir), compared to intermediate acting neutral protamine Hagedron. **Figure S14.** Funnel plot for perinatal mortality as an outcome of interest in women with gestational diabetes receiving long-acting insulin analogues (glargine and/or detemir), compared to intermediate acting neutral protamine Hagedron. **Figure S15.** Funnel plot for admission to NICU as an outcome of interest in women with gestational diabetes receiving long-acting insulin analogues (glargine and/or detemir), compared to intermediate acting neutral protamine Hagedron. **Figure S16.** Funnel plot for neonatal hypoglycaemia as an outcome of interest in women with gestational diabetes receiving long-acting insulin analogues (glargine and/or detemir), compared to intermediate acting neutral protamine Hagedron. **Figure S17.** Funnel plot for APGAR score less than 7 as an outcome of interest in women with gestational diabetes receiving long-acting insulin analogues (glargine and/or detemir), compared to intermediate acting neutral protamine Hagedron. **Figure S18.** Funnel plot for neonatal hyperbilirubinemia as an outcome of interest in women with gestational diabetes receiving long-acting insulin analogues (glargine and/or detemir), compared to intermediate acting neutral protamine Hagedron. **Figure S19.** Neonatal outcomes in women with gestational diabetes receiving glargine, compared to intermediate acting neutral protamine Hagedron. **Figure S20.** Neonatal outcomes (continued) in women with gestational diabetes receiving glargine, compared to intermediate acting neutral protamine Hagedron. **Figure S21.** Gestational age (in weeks) and birth weight (in grams) in new-borns with mother having gestational diabetes and receiving glargine, compared to intermediate acting neutral protamine Hagedron. **Figure S22.** Neonatal outcomes in women with gestational diabetes receiving detemir, compared to intermediate acting neutral protamine Hagedron. **Figure S23.** Neonatal outcomes (continued) in women with gestational diabetes receiving detemir, compared to intermediate acting neutral protamine Hagedron. **Figure S24.** Gestational age (in weeks) and birth weight (in grams) in new-borns with mother having gestational diabetes and receiving detemir, compared to intermediate acting neutral protamine Hagedron.

## Data Availability

Data sharing is not applicable to this article as no datasets were generated or analysed during the current study.

## Abbreviations

NPH: Neutral protamine Hagedron
GDM: Gestational diabetes mellitus
