# Supplementary material for: Maternal and neonatal outcomes with the use of long acting, compared to intermediate acting basal insulin (NPH) for managing diabetes during pregnancy: a systematic review and meta-analysis
Source: Diabetol Metab Syndr. 2022 Oct 21;14:154. doi: 10.1186/s13098-022-00925-7 (PMC9585834; doi:10.1186/s13098-022-00925-7)
Supplement: Supplementary file 1 — Additional file 1: Table S1. Search strategy for identification of studies to be included in the review. Table S2. Certainty of pooled estimates assessed using GRADE criteria. Table S3. Author’s judgements about study quality using the adapted Ottawa-Newcastle Risk of Bias Assessment tool. Figure S1. Risk of bias summary: review authors’ judgements about each risk of bias item for each included randomised controlled study. Figure S2. Risk of bias graph: review authors’ judgements about each risk of bias item presented as percentages across all included randomised controlled studies. Figure S3. Funnel plot for maternal hypoglycaemia as an outcome of interest in women with gestational diabetes receiving long-acting insulin analogues (glargine and/or detemir), compared to intermediate acting neutral protamine Hagedron. Figure S4. Funnel plot for caesarean delivery as an outcome of interest in women with gestational diabetes receiving long-acting insulin analogues (glargine and/or detemir), compared to intermediate acting neutral protamine Hagedron. Figure S5. Funnel plot for hypertensive disorder as an outcome of interest in women with gestational diabetes receiving long-acting insulin analogues (glargine and/or detemir), compared to intermediate acting neutral protamine Hagedron. Figure S6. Maternal outcomes in women with gestational diabetes receiving glargine, compared to intermediate acting neutral protamine Hagedron. Figure S7. Maternal gestational weight gain (Kg) and glycosylated haemoglobin (HbA1c, %) in women with gestational diabetes receiving glargine, compared to intermediate acting neutral protamine Hagedron. Figure S8. Maternal outcomes in women with gestational diabetes receiving detemir, compared to intermediate acting neutral protamine Hagedron. Figure S9. Maternal gestational weight gain (Kg) and glycosylated haemoglobin (HbA1c, %) in women with gestational diabetes receiving detemir, compared to intermediate acting neutral protamine Hagedron. Figure [file 13098_2022_925_MOESM1_ESM.doc]

**Supplementary table 1. Search strategy for identification of studies to be included in the review**

| **Search strategy for PubMed**  #1 (intermediate acting insulin OR long acting insulin OR detemir OR glargine OR degludec OR neutral protamine Hagedorn OR NPH)  #2 (pregnancy outcome OR maternal outcome OR foetal outcome OR neonatal outcome OR perinatal outcome OR delivery outcome OR adverse birth outcomes)  #3 (randomized controlled trial OR observational OR retrospective cohort OR prospective cohort OR case control)  #4 (#1 AND #2 AND #3)  #5 (Addresses[ptyp] OR Autobiography[ptyp] OR Bibliography[ptyp] OR Biography[ptyp] OR pubmed books[filter] OR Case Reports[ptyp] OR Congresses[ptyp] OR Consensus Development Conference[ptyp] OR Directory[ptyp] OR Duplicate Publication[ptyp] OR Editorial[ptyp] OR Systematic reviews OR Meta analysis OR Festschrift[ptyp] OR Guideline[ptyp] OR In Vitro[ptyp] OR Interview[ptyp] OR Lectures [ptyp] OR Legal Cases[ptyp] OR News[ptyp] OR Newspaper Article[ptyp] OR Personal Narratives [ptyp] OR Portraits[ptyp] OR Retracted Publication[ ptyp] OR Twin Study[ptyp] OR Video-Audio Media[ptyp])  #6 (#4 NOT #5)  **Search strategy in Scopus**  #1 (insulin* or intermediate acting* or long acting* or detemir or glargine or degludec or NPH or neutral protamine Hagedorn) OR TITLE-ABS-KEY (“insulin”) OR TITLE-ABS-KEY (“long-acting insulin”) OR TITLE-ABS-KEY (“intermediate acting insulin”)  #2 (randomized * or observational or cohort or cross-sectional or case-control)  #3 (Birth outcome or foetal outcome or pregnancy* or adverse birth* or complicat*) OR TITLE-ABS-KEY (“pregnancy outcome”) OR TITLE-ABS-KEY (“foetal outcome”) OR TITLE-ABS-KEY (“birth outcome”)  #1 AND #2 AND #3  **Search strategy in Cochrane Central Register of Controlled Trials (CENTRAL)**  #1 “insulin” OR “intermediate acting insulin” OR “intermediate-acting insulin” OR “long acting insulin” OR “long-acting insulin” OR “NPH” OR “neutral protamine Hagedorn” OR “detemir” OR “glargine” OR “degludec” OR “hypoglycemic agents” OR “hypoglycaemic agents” OR “hypoglycaemic drugs” OR “hypoglycemic drugs”  #2 “pregnancy” OR “birth outcomes” OR “pregnancy outcomes” OR “adverse birth outcomes” OR “adverse foetal outcomes” OR “foetal outcomes” OR “fetal outcomes” OR “maternal outcomes” OR “complications” OR “gestational outcomes”  #1 AND #2 |
| --- |

Supplementary table 2. Certainty of pooled estimates assessed using GRADE criteria

| **Outcome** | **Number of studies (type of studies)** | **Risk of bias** | **Inconsistency** | **Indirectness** | **Imprecision** | **Certainty of the evidence**  **(GRADE)** |
| --- | --- | --- | --- | --- | --- | --- |
| **Maternal outcomes** | | | | | |  |
| Hypoglycaemia | 13 (Both RCT and observational) | Serious | Not Serious | Not serious | Serious | ⨁◯◯◯ Very low |
| Hypertension | 14 (Both RCT and observational) | Serious | Not Serious | Not Serious | Serious | ⨁◯◯◯ Very low |
| Caesarean delivery | 13 (Both RCT and observational) | Serious | Not Serious | Not serious | Not Serious | ⨁◯◯◯ Very low |
| Endometritis | 2 (Both RCT and observational) | Serious | Not Serious | Serious | Serious | ⨁◯◯◯ Very low |
| Wound infection/dehiscence | 3 (Both RCT and observational) | Serious | Serious | Serious | Serious | ⨁◯◯◯ Very low |
| Gestational weight gain (Kg) | 11 (Both RCT and observational) | Serious | Serious | Not Serious | Serious | ⨁◯◯◯ Very low |
| HbA1c (%, 1^st^ trimester) | 5 (only observational) | Serious | Serious | Not Serious | Serious | ⨁◯◯◯ Very low |
| HbA1c (%, 2^nd^ trimester) | 4 (only observational) | Serious | Not serious | Serious | Not Serious | ⨁◯◯◯ Very low |
| HbA1c (%, 3^rd^ trimester) | 12 (Both RCT and observational) | Serious | Not serious | Not serious | Not serious | ⨁◯◯◯ Very low |
| **Neonatal outcomes** | | | | | | |
| Prematurity | 9 (Both RCT and observational) | Serious | Not Serious | Not serious | Serious | ⨁◯◯◯ Very low |
| Large for gestational age | 12 (Both RCT and observational) | Serious | Not Serious | Not serious | Serious | ⨁◯◯◯ Very low |
| Shoulder dystocia | 5 (Both RCT and observational) | Serious | Not Serious | Not Serious | Serious | ⨁◯◯◯ Very low |
| Small for gestational age | 4 (Both RCT and observational) | Serious | Not Serious | Serious | Serious | ⨁◯◯◯ Very low |
| Perinatal mortality | 5 (Both RCT and observational) | Serious | Not Serious | Not Serious | Serious | ⨁◯◯◯ Very low |
| Spontaneous abortion | 3 (Both RCT and observational) | Serious | Not Serious | Serious | Serious | ⨁◯◯◯ Very low |
| Congenital malformation | 8 (Both RCT and observational) | Serious | Not Serious | Not Serious | Serious | ⨁◯◯◯ Very low |
| Admission to NICU | 11 (Both RCT and observational) | Serious | Not Serious | Not Serious | Not Serious | ⨁◯◯◯ Very low |
| Respiratory distress | 11 (Both RCT and observational) | Serious | Not Serious | Not Serious | Serious | ⨁◯◯◯ Very low |
| Hypoglycaemia | 14 (Both RCT and observational) | Serious | Not Serious | Not Serious | Serious | ⨁◯◯◯ Very low |
| APGAR <7 | 8 (Both RCT and observational) | Serious | Not Serious | Not Serious | Serious | ⨁◯◯◯ Very low |
| Hyperbilirubinemia | 11 (Both RCT and observational) | Serious | Not Serious | Not Serious | Serious | ⨁◯◯◯ Very low |
| Neonatal sepsis | 3 (Only observational) | Serious | Not Serious | Serious | Serious | ⨁◯◯◯ Very low |
| Gestational age (weeks) | 14 (Both RCT and observational) | Serious | Serious | Not Serious | Serious | ⨁◯◯◯ Very low |
| Birth weight (grams) | 14 (Both RCT and observational) | Serious | Serious | Not Serious | Serious | ⨁◯◯◯ Very low |

**Downgraded for: serious indirectness (small number of pooled studies (<5) or pooled sample size of <500); serious imprecision (wide confidence interval or the estimates cross the null value); Downgraded for serious inconsistency (I^2^ value of >40%)**

**Supplementary table 3. Author’s judgements about study quality using the adapted Ottawa-Newcastle Risk of Bias Assessment tool**

|  | Chico et al (2016) | Bartal et al (2020) | Sleeman et al (2019) | Cianni et al (2008) | Egerman et al (2009) | Fang et al (2009) | Imbergamo et al (2008) | Negrato et al (2010) | Poyhonen-Alho et al (2007) | Price et al (2007) | Smith et al (2009) | Imbergamo et al (2012) |
| --- | --- | --- | --- | --- | --- | --- | --- | --- | --- | --- | --- | --- |
| Representativeness/appropriateness of participant selection  Random or consecutive recruitment=Y  Convenience sample=N  Not reported or unclear | Y | Y | Y | Y | Y | Y | Y | Y | Y | Y | Y | Y |
| Control for baseline differences in cohorts  Similarity of groups at baseline or adjustment in analyses=Y  No attempt to control or adjust=N  Not reported=NR | Y | Y | Y | Y | Y | Y | Y | Y | Y | Y | Y | Y |
| Loss to follow-up  Explanation provided for loss of participants and/or intention to treat=Y  No explanation =N | Y | Y | Y | Y | Y | Y | N | Y | Y | Y | Y | N |
| Masking of exposure to outcomes assessor  Description of masking=Y  No masking or no description =N | Y | Y | Y | Y | Y | Y | Y | Y | Y | Y | Y | Y |
| Ascertainment of condition  Description of ascertainment/diagnostic criteria=Y  No description or patient self-report=N | Y | Y | Y | Y | Y | Y | Y | N | Y | Y | Y | Y |
| Documentation of other treatment modalities  Documentation=Y  No documentation=N | Y | Y | Y | Y | N | Y | Y | Y | Y | Y | Y | Y |
| Extent to which valid outcomes are described  Adequate description of outcome=Y  Insufficient detail regarding outcome or follow-up time=N | Y | Y | Y | Y | Y | Y | N | Y | N | Y | Y | Y |
| Prespecification of harms, mode of harms collection  Description of a list of harms assessed or monitoring=Y  No such description or passive harms collection=N  No adverse events reported=NA | Y | Y | Y | Y | Y | Y | Y | Y | Y | N | Y | Y |
| Financial Conflict of interest (COI)  Funding source reported=Y  Funding source not reported=N | Y | Y | Y | N | Y | Y | Y | Y | N | Y | Y | Y |

**Supplementary figure 1. Risk of bias summary: review authors’ judgements about each risk of bias item for each included randomised controlled study.**


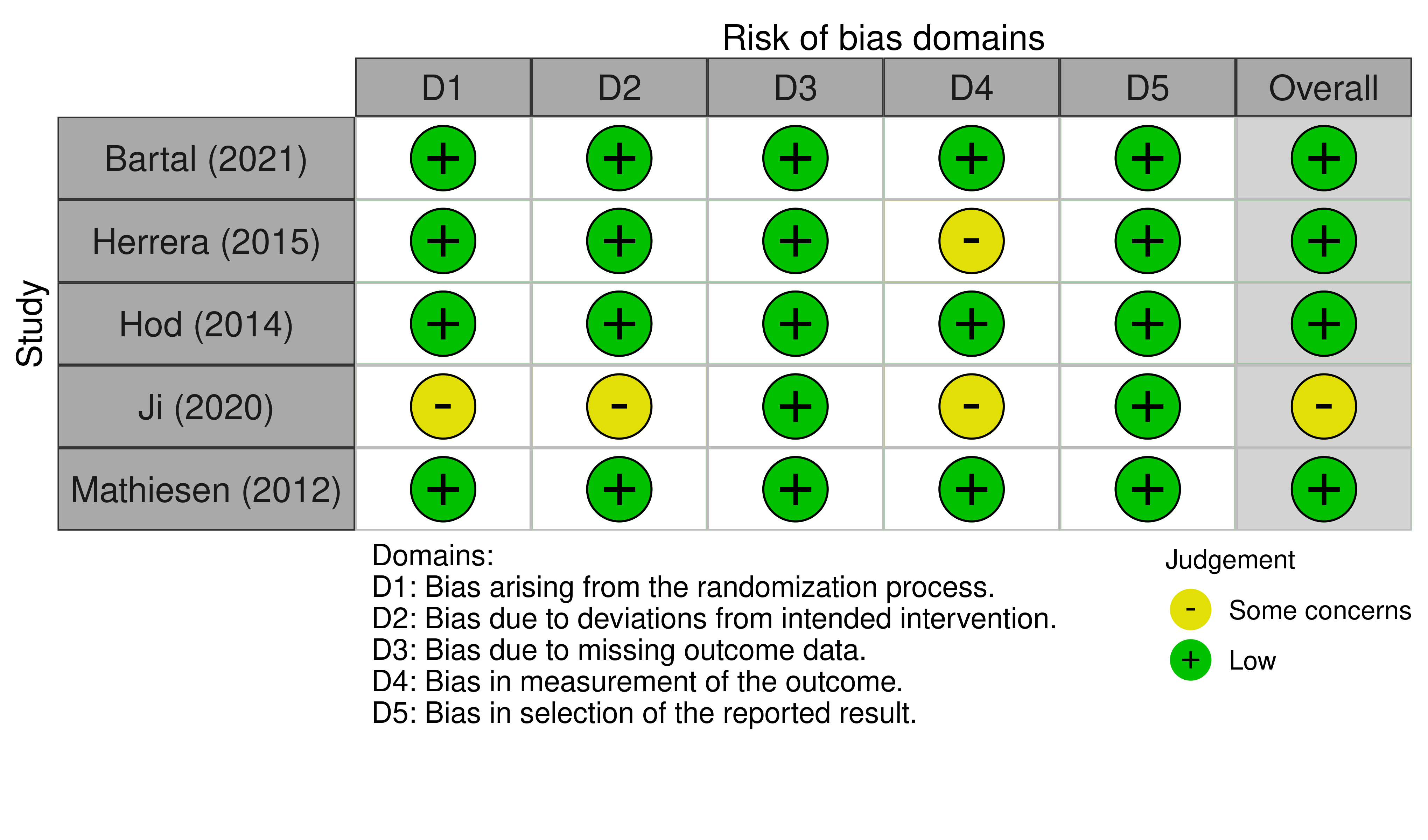


**Supplementary figure 2. Risk of bias graph: review authors’ judgements about each risk of bias item presented as percentages across all included randomised controlled studies**


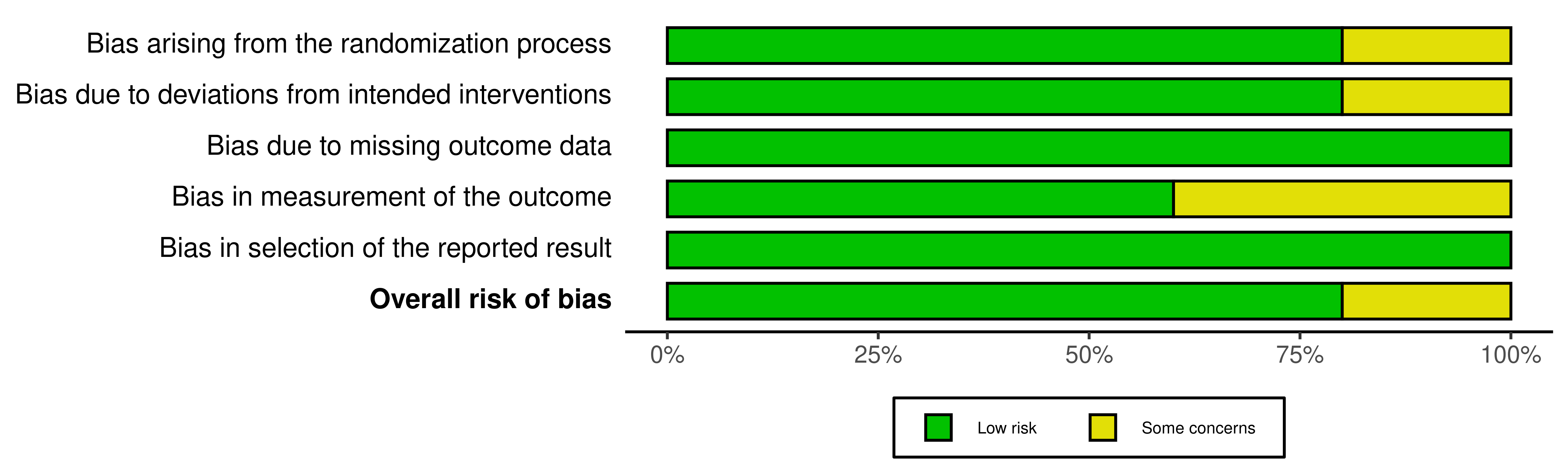


**Supplementary figure 3. Funnel plot for maternal hypoglycaemia as an outcome of interest in women with gestational diabetes receiving long-acting insulin analogues (glargine and/or detemir), compared to intermediate acting neutral protamine Hagedron**

**Supplementary figure 4. Funnel plot for caesarean delivery as an outcome of interest in women with gestational diabetes receiving long-acting insulin analogues (glargine and/or detemir), compared to intermediate acting neutral protamine Hagedron**

**Supplementary figure 5. Funnel plot for hypertensive disorder as an outcome of interest in women with gestational diabetes receiving long-acting insulin analogues (glargine and/or detemir), compared to intermediate acting neutral protamine Hagedron**

**Supplementary figure 6. Maternal outcomes in women with gestational diabetes receiving glargine, compared to intermediate acting neutral protamine Hagedron**

**Supplementary figure 7. Maternal gestational weight gain (Kg) and glycosylated haemoglobin (HbA1c, %) in women with gestational diabetes receiving glargine, compared to intermediate acting neutral protamine Hagedron**

**Supplementary figure 8. Maternal outcomes in women with gestational diabetes receiving detemir, compared to intermediate acting neutral protamine Hagedron**

**Supplementary figure 9. Maternal gestational weight gain (Kg) and glycosylated haemoglobin (HbA1c, %) in women with gestational diabetes receiving detemir, compared to intermediate acting neutral protamine Hagedron**

**Supplementary figure 10. Funnel plot for preterm birth as an outcome of interest in women with gestational diabetes receiving long-acting insulin analogues (glargine and/or detemir), compared to intermediate acting neutral protamine Hagedron**

**Supplementary figure 11. Funnel plot for large for gestational age as an outcome of interest in women with gestational diabetes receiving long-acting insulin analogues (glargine and/or detemir), compared to intermediate acting neutral protamine Hagedron**

**Supplementary figure 12. Funnel plot for small for gestational age as an outcome of interest in women with gestational diabetes receiving long-acting insulin analogues (glargine and/or detemir), compared to intermediate acting neutral protamine Hagedron**

**Supplementary figure 13. Funnel plot for congenital malformation as an outcome of interest in women with gestational diabetes receiving long-acting insulin analogues (glargine and/or detemir), compared to intermediate acting neutral protamine Hagedron**

**Supplementary figure 14. Funnel plot for perinatal mortality as an outcome of interest in women with gestational diabetes receiving long-acting insulin analogues (glargine and/or detemir), compared to intermediate acting neutral protamine Hagedron**

**Supplementary figure 15. Funnel plot for admission to NICU as an outcome of interest in women with gestational diabetes receiving long-acting insulin analogues (glargine and/or detemir), compared to intermediate acting neutral protamine Hagedron**

**Supplementary figure 16. Funnel plot for neonatal hypoglycaemia as an outcome of interest in women with gestational diabetes receiving long-acting insulin analogues (glargine and/or detemir), compared to intermediate acting neutral protamine Hagedron**

**Supplementary figure 17. Funnel plot for APGAR score less than 7 as an outcome of interest in women with gestational diabetes receiving long-acting insulin analogues (glargine and/or detemir), compared to intermediate acting neutral protamine Hagedron**

**Supplementary figure 18. Funnel plot for neonatal hyperbilirubinemia as an outcome of interest in women with gestational diabetes receiving long-acting insulin analogues (glargine and/or detemir), compared to intermediate acting neutral protamine Hagedron**

**Supplementary figure 19. Neonatal outcomes in women with gestational diabetes receiving glargine, compared to intermediate acting neutral protamine Hagedron**

**Supplementary figure 20. Neonatal outcomes (continued) in women with gestational diabetes receiving glargine, compared to intermediate acting neutral protamine Hagedron**

**Supplementary figure 21. Gestational age (in weeks) and birth weight (in grams) in new-borns with mother having gestational diabetes and receiving glargine, compared to intermediate acting neutral protamine Hagedron**

**Supplementary figure 22. Neonatal outcomes in women with gestational diabetes receiving detemir, compared to intermediate acting neutral protamine Hagedron**

**Supplementary figure 23. Neonatal outcomes (continued) in women with gestational diabetes receiving detemir, compared to intermediate acting neutral protamine Hagedron**

**Supplementary figure 24. Gestational age (in weeks) and birth weight (in grams) in new-borns with mother having gestational diabetes and receiving detemir, compared to intermediate acting neutral protamine Hagedron**
